# Cancer of the Stomach

**Published:** 1908-06

**Authors:** 


**Cancer of the Stomach.** By A. W. MAYO ROBSON, D.Sc., F.R.C.S. Pp. 218. London: James Nisbet & Co., Ltd. 1907.

This book forms one of the *Nisbet Modern Clinic Series*, and gives a succinct account of the subject, which will be found useful to the practitioner. It is admittedly wanting in much that is now known of the pathology of cancer of the stomach, and nearly half the book is taken up with descriptions of the various operations for the cure and relief of malignant stomach tumours that could be equally well obtained from works on operative surgery. To those who are familiar with the recent advances in gastric surgery and pathology it will not appeal, but to many who desire a clear and not too detailed summary of the present knowledge of the disease of which it treats the book will be welcome, and its value is enhanced by a liberal supply of helpful illustrations. The occurrence of cancer of the stomach is more common than is popularly supposed, and a greater familiarity with the symptoms and operative possibilities in such cases is desirable for the general practitioner, in order that he may seek the advice of the practical surgeon in an early stage of the disease, in which alone a radical operation is possible with any degree of success.

